# Reactive Oxygen Species-Responsive Nanococktail With Self-Amplificated Drug Release for Efficient Co-Delivery of Paclitaxel/Cucurbitacin B and Synergistic Treatment of Gastric Cancer

**DOI:** 10.3389/fchem.2022.844426

**Published:** 2022-03-04

**Authors:** Lijun Pang, Lei Zhang, Hong Zhou, Ling Cao, Yueqin Shao, Tengyun Li

**Affiliations:** ^1^ Department of Oncology, The Affiliated Jiangsu Shengze Hospital of Nanjing Medical University, Suzhou, China; ^2^ Department of Pharmacy, The Affiliated Jiangsu Shengze Hospital of Nanjing Medical University, Suzhou, China

**Keywords:** nanococktail, polymeric prodrug, combination therapy, ROS-responsive biodegradability, self-amplifiable drug release

## Abstract

Application of drug combinations is a powerful strategy for the therapy of advanced gastric cancer. However, the clinical use of such combinations is greatly limited by the occurrence of severe systemic toxicity. Although polymeric-prodrug-based nanococktails can significantly reduce toxicity of drugs, they have been shown to have low intracellular drug release. To balance between efficacy and safety during application of polymeric-prodrug-based nanococktails, a reactive oxygen species (ROS)-responsive nanococktail (PCM) with self-amplification drug release was developed in this study. In summary, PCM micelles were co-assembled from ROS-sensitive cucurbitacin B (CuB) and paclitaxel (PTX) polymeric prodrug, which were fabricated by covalently grafting PTX and CuB to dextran *via* an ROS-sensitive linkage. To minimize the side effects of the PCM micelles, a polymeric-prodrug strategy was employed to prevent premature leakage. Once it entered cancer cells, PCM released CuB and PTX in response to ROS. Moreover, the released CuB further promoted ROS generation, which in turn enhanced drug release for better therapeutic effects. *In vivo* antitumor experiments showed that the PCM-treated group had lower tumor burden (tumor weight was reduced by 92%), but bodyweight loss was not significant. These results indicate that the developed polymeric prodrug, with a self-amplification drug release nanococktail strategy, can be an effective and safe strategy for cancer management.

## Introduction

In 2020, over 1,080,000 new cases of gastric cancer (GC) were diagnosed and 768,000 mortalities were reported making it the sixth most common cancer and the third cause of cancer-related deaths in the world (Sung et al., 2021). Chemotherapy remains the major treatment strategy among the various therapeutic strategies for gastric cancer (Ma et al., 2021). However, due to the physiological complexity and drug resistance of GCs, the treatment of GC with a single drug or even a stand-along therapy strategy has not been sufficient enough for inhibition of tumor proliferation (Shafabakhsh et al., 2020; Zhang Z. et al., 2020).

It has been reported that the chemotherapy strategy which utilizes a combination of multiple drugs, or the so-called “drug cocktail,” can achieve a synergistic effect on the tumor cells (Lang et al., 2019). Furthermore, the drug cocktail maximizes the therapeutic effect through different signaling pathways, leading to higher therapeutic outcome and low side effects than the single chemotherapeutic drug strategy (Hu et al., 2016). However, various challenges of conventional combination therapy, such as different pharmacokinetics of the combined drugs, lack of tumor targeting, and unwanted side effects, have greatly hindered the application of the drug cocktail strategy in clinical practice (Qi et al., 2017).

Benefiting from the development of nanotechnology, the nanococktail strategy is developed by loading different drugs into a single nanocarrier. The strategy can effectively deliver therapeutic agents to the targeted sites with similar pharmacokinetics and reduced toxicity (Fang et al., 2018). The conventional nanococktails are prepared by encapsulating several drugs into a specific nanocarrier, such as polymeric micelles, liposome, and mesoporous silica nanoparticles (Meng et al., 2015; Zununi Vahed et al., 2017; Zhao et al., 2019). Although these nanococktails reduce the side effects and exhibit the synergistic effect in tumor therapy, premature drug release could potentially deteriorate the already compromised health condition of patients with the tumor (Li et al., 2019). Moreover, because of the different loading potentials against different drug compounds, the ratio of drugs in the nanococktail may not be easily modulated according to their required concentration (Chen et al., 2019).

Polymeric-prodrug-based nanomedicine involves conjugation of multiple drugs to a polymer through covalent bonds, such as thioketal (TK), disulfide, and ester bonds (Huang et al., 2017; Hao et al., 2020; Lu et al., 2020). Recently, nanomedicine has received great attention of researchers because it reduces the induced harm caused by premature drug release (Zhang et al., 2019; Zhang et al., 2021). When these nanoparticles reach the tumor tissue, the linkage between drug and the polymer is cleaved in response to endogenous (pH, reduction, reactive oxygen species (ROS), and enzyme) and exogenous (light and magnetic field) stimuli to selectively release the drugs (Zhang et al., 2019; Zhang et al., 2021). Therefore, the drug-related side effects can be significantly minimized because specific conjugated drugs can only be released after the cleave of the linker upon reaching the specific tumor tissue (Sui et al., 2019). However, the selectively and efficiency of stimuli-triggered drug release from the nanococktails are remarkably decreased due to the heterogeneity of different tumors (Ye et al., 2017). The benefits of polymeric-prodrug-based nanococktails are, hence, left with a catch-22 situation between their efficacy and safety (Sui et al., 2019). In the present study, a simple ROS-sensitive nanococktail was developed to improve drug release efficiency and selectively, enhance therapeutic efficacy, and reduce the side effects of polymeric-prodrug-based nanococktail drugs. Generally, it was evident that the developed nanococktail amplified the level of ROS in cancer cells, accelerated the drug release, and thus, broke the catch-22 limitation of nanococktail strategy.

Cucurbitacin B (CuB) is a typical tetracyclic triterpenoid compound, which exists widely in the plant kingdom (Garg et al., 2018). Accumulating evidence has shown that CuB can increase intracellular ROS level, hence inhibiting the growth of GC as well as colon, breast, and lung cancer cells (Yasuda et al., 2010; Ren et al., 2015; Luo et al., 2018; Xu et al., 2020). According to Wang et al. (2020) and Li et al. (2021), co-CuB can effectively enhance the level of ROS in cancer cells and, hence, accelerate and amplify degradation of ROS-responsive prodrugs. Therefore, co-delivery CuB for increasing intracellular concentration of ROS in cancer cells could greatly improve the efficacy of ROS-responsive nanococktail. Furthermore, CuB can potentiate the antitumor effect of numerous chemotherapiutic drugs, such as paclitaxel (PTX), methotrexate, and gemcitabine (Thoennissen et al., 2009; Lee et al., 2011; Marostica et al., 2017). Therefore, CuB-based nanococktail increases the ROS to promote drug release in cancer cells and also achieve a synergistic effect for the treatment of GC.

**SCHEME 1:**
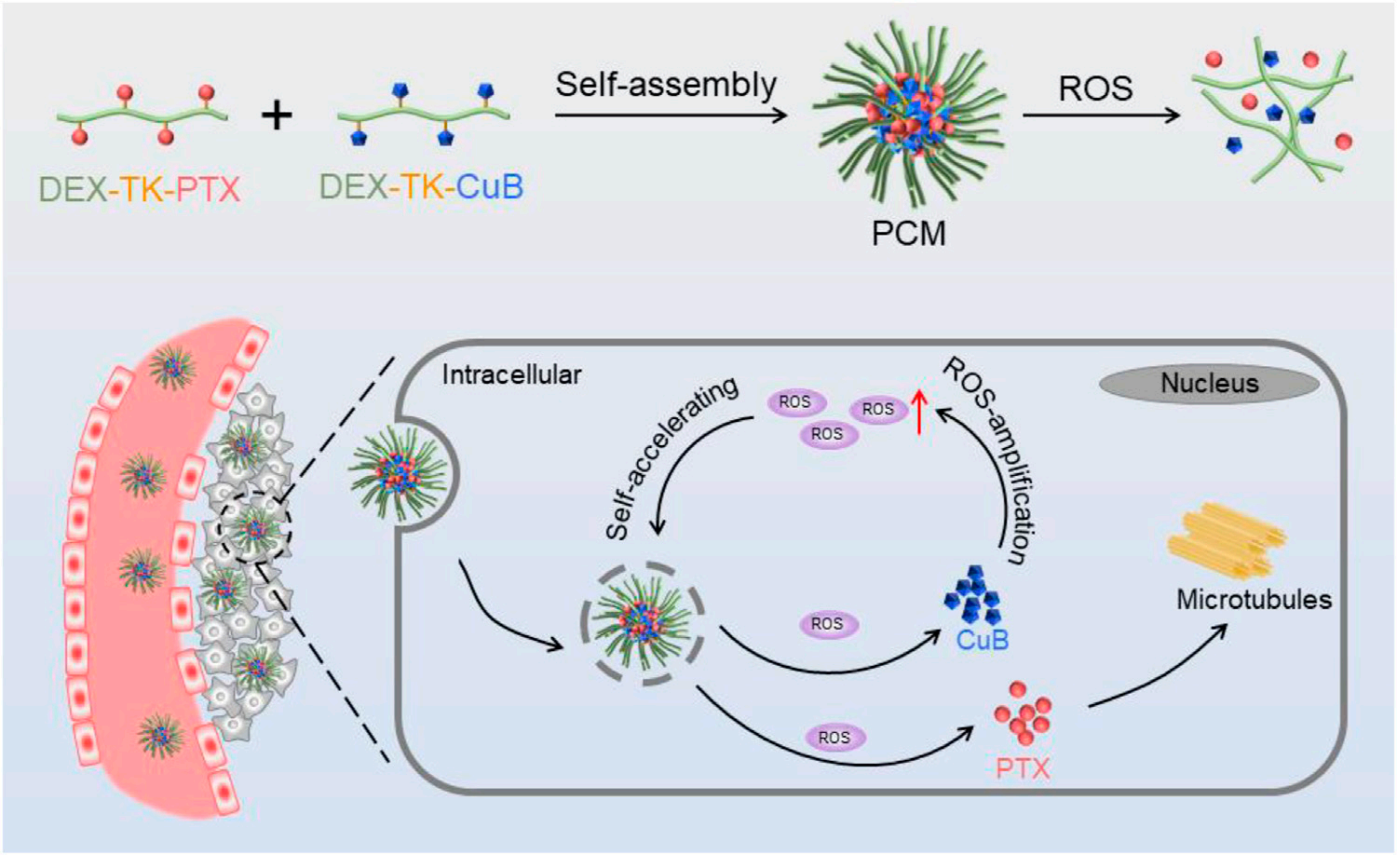
Schematic illustration of the preparation and intracellular performance of PCM nanococktails.

In the present study, a self-amplification release nanococktail (PCM) was developed by self-assembling of both ROS-responsive CuB (DEX-TK-CuB) and PTX (DEX-TK-PTX) polymeric prodrugs (Scheme 1). The two prodrugs were prepared by conjugating PTX and CuB to the hydrophilic dextran through an ROS-sensitive linkage several times. Reactive oxygen species (ROS)-responsive CuB (DEX-TK-CuB) and DEX-TK-PTX can co-assembly into micelles in an aqueous solution to form a self-amplification release nanococktail (PCM). Therefore, the side effect of CuB and PTX could significantly reduce because TK is very stable in the absence ROS. After entering the cancer cells, the TK linkage can be triggered by the endogenous ROS to break and release CuB and PTX. The released CuB could further induce massive generation of ROS, which then accelerates and amplifies release of more drugs. Therefore, the rapid and complete release of the drug ultimately enhances the efficiency of the nanococktail in the treatment of cancer.

## Materials and Experiments

### Synthesis of Thioketal-Paclitaxel and Thioketal-Cucurbitacin B

The ROS-cleavable thioketal linker (TK) was first fabricated as described in the previous studies (Chen et al., 2016; Hu et al., 2017). In brief, 6.8 and 6.0 g of anhydrous acetone and anhydrous 3-mercaptopropionic acid, respectively, were mixed and stained with dry hydrogen chloride at room temperature for 6 h. At the end of the reaction, the mixture was cooled with an ice–salt mixture for crystallization. Thereafter, the crystals were filtered and washed with abundant hexane and cold water. The product was obtained after drying under vacuum (yield: 44.5%). The detection of molecular weight (MW) of TK was determined by mass spectrometry, and the MW was found to be 251.08, calculated to 251.05.

Subsequently, to obtain TK-TPX or TK-CuB, the prepared TK was conjugated to PTX or CuB, respectively. In brief, 4 mmol of TK was dissolved in 6 ml of acetic anhydride and stirred at room temperature in nitrogen atmosphere for 2 h. The solvent was then removed under reduced pressure and further dried under vacuum to obtain TK anhydride. Subsequently, all the TK anhydride, 4 mmol PTX, and 0.4 mmol DMAP were dissolved in dry DMSO, and the mixture was stirred in nitrogen atmosphere at room temperature for 14 h. The reaction product was purified through silica gel column chromatography using CH_2_Cl_2_ and ethyl acetate (3/1, v/v) as the eluent. A white solid (TK-PTX) was obtained with an yield of 71.3%. TK-CuB was also prepared using the same method by just changing the PTX with CuB (yield 73.5%).

### Synthesis of DEX-Thioketal-Paclitaxel and DEX-Thioketal-Cucurbitacin B

TK-PTX and TK-CuB were conjugated to DEX though an ester reaction between prodrugs and DEX to generate DEX-TK-PTX or DEX-TK-CuB, respectively. In this study, the protocol of DEX-TK-PTX was described to present the polymeric prodrug synthesis details. Typically, the TK-PTX (108.8 mg, 0.1 mmol), EDC (28.8 mg, 0.15 mmol), DMAP (18.3 mg, 0.15 mmol), and DEX (3.5 g, 0.05 mmol) were dissolved in 40 ml anhydrous DMSO and stirred under nitrogen atmosphere at room temperature. After 24 h of reaction, the solution was transferred into a dialysis bag (MWCO: 1.5 kDa) and was dialyzed against DMSO for 24 h and then into distilled water for 48 h. Finally, the solution was lyophilized to obtain DEX-TK-PTX (yield = 65.4%). The DEX-TK-CuB was obtained using the same synthesis route with the yield of 61.7%.

The drug content in the polymeric prodrugs was detected using UV spectrophotometry using a standard curve method. The drug content was calculated using the following equation:

$$\mathrm{Drug}\;\mathrm{content}\left(100\%\right)=\frac{\text{Mass of drug in prodrug}}{\text{Mass of prodrug}}\times 100\%.$$

#### Micelles Preparation

A simple dialysis method was used to prepare the combination prodrug micelles. Typically, 2 mg of prodrugs (or a mixture of different prodrugs) was dissolved in 0.1 ml of DMSO. The prodrug solution was then dropped into 1 ml water under violent stirring. After 2 h stirring, the mixture was dialyzed against PBS in a Spectra/Por dialysis tube (MWCO: 3.5 kDa) at 4°C for 24 h to eliminate DMSO. Finally, the micelles were filtered through 0.45 μm syringe filters and stored at 4 °C. Furthermore, using the similar method, **P**TX and **C**uB co-loaded **m**icelles (denoted PCM), only **P**TX-loaded **m**icelles (denoted as PM), and only **C**uB-loaded **m**icelles (named as CM) were obtained.

For coumarin-6-loaded micelle preparation, 10 μg of coumarin-6 and 2 mg of prodrugs were dissolved in DMSO and then prepared as described earlier.

The drug loading content (DLC) of PTX and CuB was detected by HPLC and calculated using the following Eq. 1:

$$\text{DLC}\left(\text{\%}\right)=\frac{\text{Mass of drug in micelles}}{\text{Mass of micelles}}\times 100\%.$$ (1)

### *In Vitro* Drug Release

A simple ultrafiltration centrifugation method was used to assay the release behaviors of PTX and CuB from PCM. The PBS (pH 7.4) containing 0.5% (w/v) Tween-80 with 0, 0.1, and 10 mM H_2_O_2_ was utilized as the release medium. In brief, freshly prepared PCM (containing 30 μg of PTX and 15 μg of CuB) was dissolved in 4 ml of release medium and cultured at 37°C with slight shaking. At pre-set time intervals, one sample was collected and centrifuged at 5,000 g for 10 min using a centrifugal filter unit (MWCO = 3.5 kDa). The UV spectrometry was used to detect the concentration of released CuB and PTX.

### Reactive Oxygen Species-Triggered Micelle Degradation

Micelles (2 mg/ml) were cultured in PBS (pH 7.4) with or without 10 mM H_2_O_2_ for 12 h at 37°C. After treatment, the size of micelles was then measured using DLS.

### Cellular Uptake

Human gastric cancer BGC-823 cells were seeded on a six-well plate with 5 × 10^4^ cells per well and incubated for 24 h. The cells were then separately treated with coumarin-6-loaded PM, CM, and PCM, with the final concentration of coumarin-6 being 400 ng/ml. After culturing for 2 or 4 h, the cells were washed with PBS, fixed by 4% paraformaldehyde, stained with Hoechst 33,342, washed with PBS, and then, directly recorded on a fluorescence microscope.

### Reactive Oxygen Species Generation in Cancer Cells

The CuB-mediated ROS generation in cancer cells was studied using a fluorescence microscope and flow cytometry. For fluorescence microscope assay, BGC823 cells were seeded on a six-well plate with 6 × 10^4^ cells per well and incubated overnight. Then, the cells were pretreated with or without 4 mmol/L of N-acetyl cysteine (NAC) for 1 h. Subsequently, cells were separately cultured with PBS, CuB, PTX, CM, PM, and PCM2 which were equal to 1.0 μg/ml of CuB for 8 h. After treatment, the cells were stained with DCFH-DA for 20 min at 37°C, washed with PBS for three times, fixed by 4% paraformaldehyde, and then, quickly observed under a fluorescence microscope.

For flow cytometry assay, the BGC823 cells were seeded on a six-well plate with 6 × 10^4^ cells per well and incubated overnight. Then, some cells were incubated with free CuB, CM, PM, and PCM2 for various concentrations (equal to 0.5, 1.0, and 2.0 μg/ml of CuB) or containing 1.0 μg/ml CuB for different times (4, 8, and 12 h), respectively. At the end of incubation, the cells were stained with DCFH-DA for 20 min at 37°C and then detected through flow cytometry.

### *In Vitro* Cytotoxicity

The BGC823 cells or human SGC7901 cells were seeded into a 96-well plate at the density of 5,000 cells per well. After cultured for 24 h, the cells were incubated with CuB, PTX, PTX+CuB, CM, PM, or PCM with different concentrations (0.0001, 0.001, 0.01, 0.1, 1, 50, 100, and 200 μg/ml, equal to PTX) for 48 h. A 20 μL of MTT solution (5 mg/ml) was then added into each well and incubated for further 3 h. Finally, the old medium was replaced with 100 μL DMSO to dissolve the formed formazan salts, and the adsorption of each well was recorded on a microplate reader at 490 nm.

### *In Vivo* Antitumor Efficacy

For BGC-823 xenograft model construction, 6 × 10^6^ cells resuspended in 200 μL of PBS solution were subcutaneously inoculated in the right flank. When the tumor volume reached about 100 mm^3^, mice were randomized into seven groups of six mice each with similar mean tumor volumes between the groups and then treated with PBS, CuB, PTX, PTX+CuB, PM, CM, and PCM at 5.0 mg/kg PTX and 1.7 mg/kg CuB. The formulations were administered through the tail vein on days 0, 3, 6, and 9, respectively, as a total for four times. The body weight, tumor length (***a***), and width (***b***) were detected every 2 days. The tumor volume was calculated as volume = 1/2 × ***a*** × ***b*** ^2^.

After 3 weeks, all mice were sacrificed to harvest the tumors which were weighted, and their tumor suppression ratio (TSR) was calculated according to the Eq. 2:

$$\text{ TSR}\left(\text{\%}\right)=\frac{\text{Tumor mass of PBS group – tumor mass of treatment group}}{\text{Tumor weigh of PBS group}}\text{×}100\text{\%}\text{.}$$ (2)

### Statistical Analysis

Each experiment in the present study was carried out triplicate. The results were presented as mean ± SD. Inferential statistics were carried out using the *t*-test or one-way analysis of variance (ANOVA) followed by Tukey’s post hoc test to compare the means. *p* < 0.05 was defined as a statistically significant difference.

## Results and Discussion

### Paclitaxel and Cucurbitacin B Conjugation Synthesis and Characterization

The DEX, a naturally water-soluble bacterial polysaccharide, has been widely used in medicine because of its excellent biocompatibility and biodegradability (Raveendran et al., 2016; Zhang X. et al., 2020). The abundance of hydroxyl groups enables DEX to be conjugated with drugs to increase the solubility, prolong the circulation time, and improve the stability of drugs (Zhang T. et al., 2020; Zeng et al., 2020). Moreover, in comparison with poly(ethylene glycol), DEX shows better stability and less tendency to nonspecific protein adsorption as a drug carrier (Li et al., 2017). Therefore, DEX is an ideal and safe biomedical material.

**FIGURE 1:**
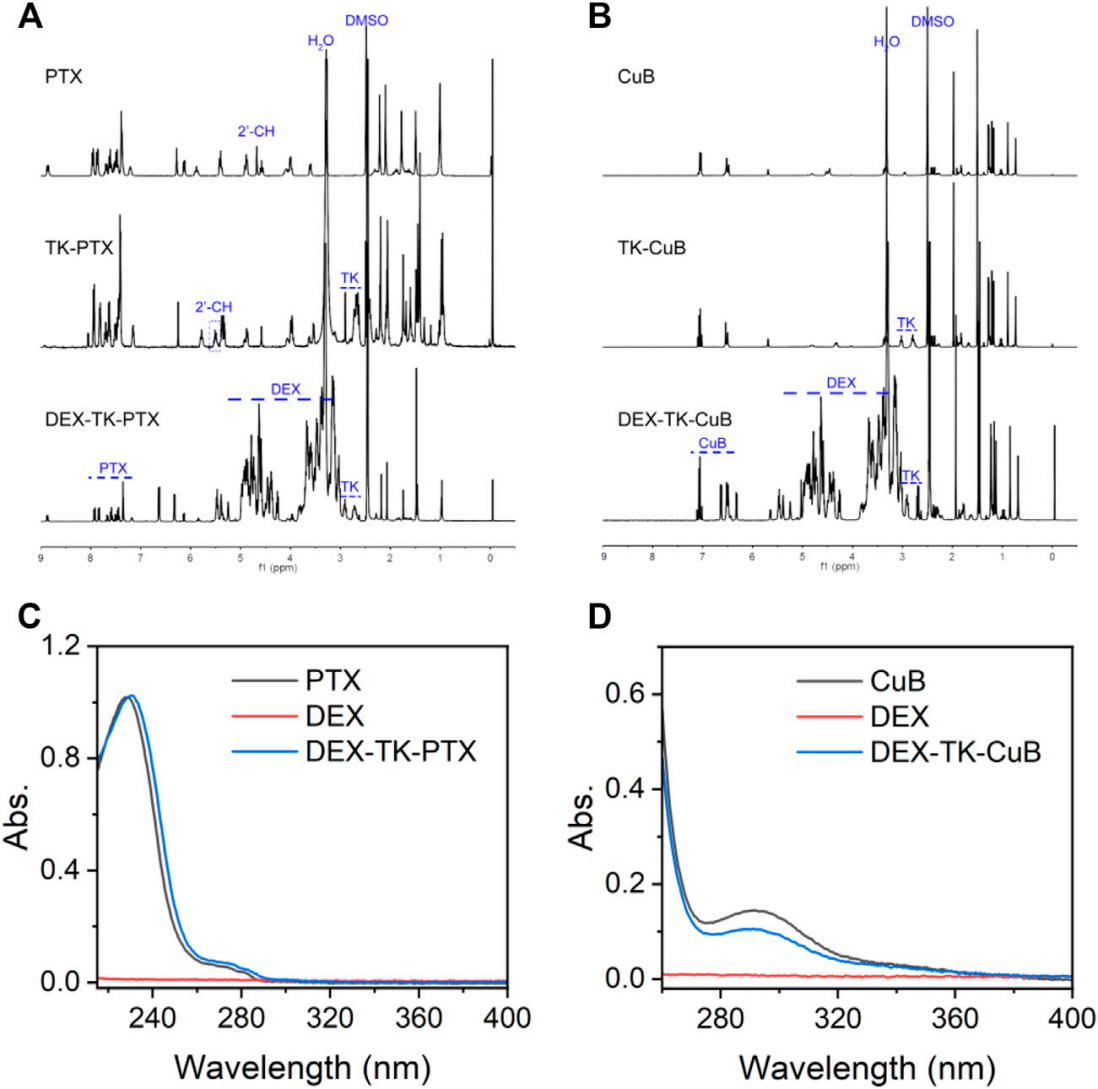
Characterization of prodrugs. **(A)** ^1^H NMR spectra of PTX, TK-PTX, and DEX-TK-PTX in DMSO-*d6*. **(B)** ^1^H NMR spectra of CuB, TK-CuB, and DEX-TK-CuB in DMSO-*d6*. **(C)** UV spectra of PTX, TK-PTX, and DEX-TK-PTX. **(D)** UV spectra of CuB, TK-CuB, and DEX-TK-CuB.

In this work, the polymeric prodrugs were prepared by modifying PTX or CuB to DEX though an ROS-sensitive TK linker *via* a two-step esterification reaction. The synthesis route was as presented in Supplementary Scheme S1. First, the ROS-cleavable TK linkage was prepared based on the previous report (Chen et al., 2016; Hu et al., 2017). The MS results were in consistence with previous reports (Chen et al., 2016; Hu et al., 2017). Subsequently, TK-modified PTX (TK-PTX) and -CuB (TK-CuB) were obtained. To increase the yield and decrease the by-product, the anhydride TK was synthesized and used instead of TK due to its high reactivity with hydroxyl groups. The structure of TK-PTX and TK-CuB were verified through ^1^H NMR (Figures 1A,B) and MS (Supplementary Figure S1). In the ^1^H NMR spectrum of TK-PTX (Figure 1A), the peaks that appeared at 2.9 and 3.1 ppm, which belong to the two methylene groups of TK, demonstrated the successful reaction between TK and PTX. Moreover, as compared with free PTX, the 2′-CH proton peaks of TK-PTX shifted from 4.7 ppm to 5.6 ppm. This suggests that the reaction between PTX and TK took place preferentially at the 2′-hydroxyl of PTX (Wang et al., 2016). Similarly, the characteristic peaks of both TK and CuB emerged in the ^1^H NMR spectrum of TK-CuB. This suggested that TK-CuB was successfully prepared (Figure 1B). In addition, the MS results of TK-PTX and TK-CuB exhibited peaks at m/z = 1,087.1 and m/z = 791.7 matching with the [M-H]^-^, respectively. This was utterly consistent with the theoretical calculation value (Supplementary Figures S1A,B). This result further demonstrated that TK-PTX and TK-CuB were successfully synthesized.

Finally, it was found that DEX reacted with TK-PTX and TK-CuB to generate the DEX-TK-PTX and DEX-TK-CuB, respectively. The ^1^H NMR spectrum of DEX-TK-PTX showed all the expected resonance peaks characteristic of PTX and DEX, such as the peak at 7.8 ppm corresponding to the protons of benzene in PTX and the CH peak at 0.55 ppm related to those of DEX (Figure 1A). A similar phenomenon could also be observed in the ^1^H NMR spectrum of DEX-TK-CuB (Figure 1B). These results indicate the successful synthesis of DEX-TK-CuB. The UV spectra of DEX, PTX, DEX-TK- PTX, CuB, and DEX-TK-CuB were presented as shown in Figures 1C,D. It was evident that the DEX had no UV absorption in the range of 250–400 nm, and both PTX and DEX-TK-PTX showed the maximum absorption at 227 nm. Similarly, it was found that the CuB and DEX-TK-CuB exhibited maximum absorption at 293 nm. The PTX and CuB content in DEX-TK-PTX and DEX-TK-CuB were 18.8 ± 1.0 and 19.5 ± 1.2%, respectively, detected using HPLC by a standard curve method.

### NPs Preparation and Characterization

This was carried out through conjugation of the hydrophobic CuB and PTX to the hydrophilic DEX to obtain amphiphilic DEX-TK-CuB and DEX-TK-PTX. The two amphiphilic polymeric prodrugs (DEX-TK-CuB and DEX-TK-PTX) provided an opportunity for self-assembly into micelles in the aqueous medium. As a proof of the conception, the CMC values (the fundamental parameter of micelles) of two prodrug micelles were determined using Nile red as the fluorescence probe. As shown in Supplementary Figures S2A,B, the CMC values of the two prodrugs were calculated to be 12.7 (DEX-TK-PTX) and 10.8 μg/ml (DEX-TK-PTX). The low CMC values of both prodrugs suggested that the excellent antidilution stability *in vivo* of the formed micelles.

Subsequently, the micelles containing PTX, CuB, and their combination were prepared using a simple dialysis method and denoted as PM, CM, and PCM, respectively. To achieve the highest antitumor efficiency, a series of PCMs with different molar ratios of PTX to CuB were prepared and named as PCM1 ∼ PCM5. PCM1 to PCM5 have a similar particle size, size distribution, and zeta potential (Supplementary Table S1). It was found that, at 48 h, PCM2 induced the highest cytotoxicity against BGC823 cells with the IC_50_ value of 2.78 ug/mL. Therefore, PCM2 (PTX/CuB = 3: 1, mass/mass) was selected as the final micelle for the following studies.

The physical properties of PM, CM, and PCM2 were measured through TEM and dynamic light scattering (DLS). The TEM images revealed that all the three micelles of PM, CM, and PCM2 had clear spherical morphology with uniform distribution. The hydrodynamic particle size of PM, CM, and PCM2 in PBS was (82.6 ± 3.4) (80.8 ± 3.0), and (78.7 ± 2.5) nm, respectively, as determined by DLS. It was found that these micelles had a narrow distribution as evidenced by those whose PDI was lower than 0.22 (Supplementary Table S1). The appropriate particle size is conducive for the accumulation of micelles in tumor tissues through the enhanced permeability and retention (EPR) effect, ultimately achieving high-efficiency drug delivery (Goos et al., 2020). Additionally, the stability of micelles in PBS (pH 7.4) or PBS (pH 7.4) containing 10% FBS was tested using the DLS method (Supplementary Figure S3). After an incubation period of 48 h, the size of PM, CM, and PCM2 had no significant changes in the two conditions of PBS (Supplementary Figure S3A) and PBS containing 10% FBS (Supplementary Figure S3B). This indicated that these micelles have an excellent stability.

### Reactive Oxygen Species-Triggered Structure Change and Drug Release

**FIGURE 2:**
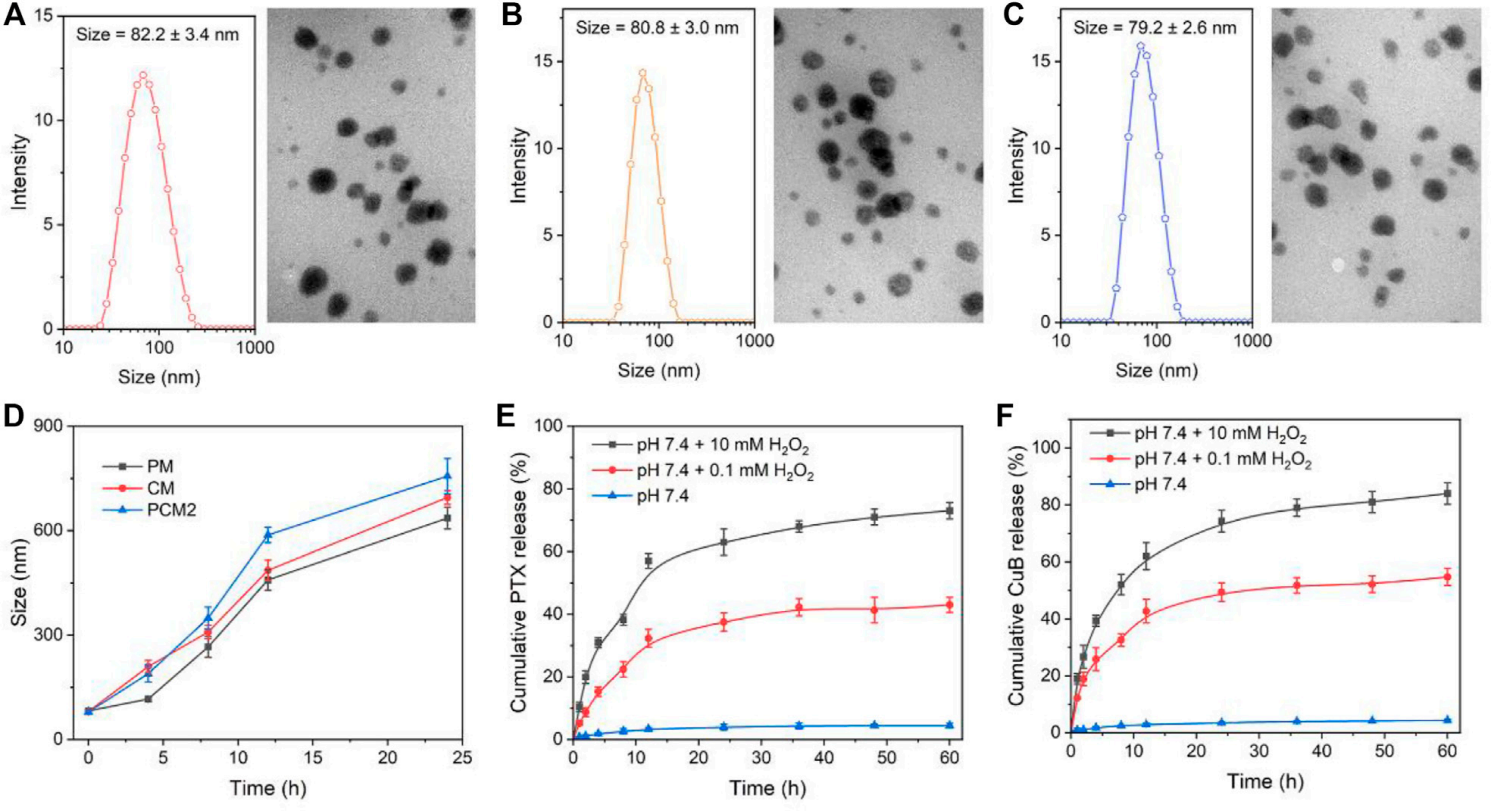
Micelle characterization. **(A–C)** Size distribution and TEM images of PM **(A)**, CM **(B)**, and PCM2 **(C)**. **(D)** Changes in size of PM, CM, and PCM2 after treatment with 10 mM H_2_O_2_ for 0, 2, 4, 8, and 16 h, respectively. **(E,F)** Cumulative release of PTX **(E)** and CuB **(F)** from PCM2 after incubation with various dose of H_2_O_2_. The data in D, E, and F are presented as the mean ± SD, *n* = 3.

In this work, the PTX and CuB was covalently conjugated to DEX through an ROS-cleavable TK linker. In the ROS environment, the TK linker would be broken, resulting in release of the modified drugs and degradation of micelles. To investigate the ROS-responsive capability of prodrug micelles, size change and drug release behavior of micelles in different ROS conditions were studied by using H_2_O_2_ as a typical ROS stimulus (Hu et al., 2017). First, the time-dependent size change of PM, CM, and PCM2 in 1 mM H_2_O_2_ was monitored by DLS. An increase in size of three micelles was observed after incubation with 1 mM H_2_O_2_ (Figure 2B). After incubation for 8 h, the average size of PM, CM, and PCM2 changed from 50, 60, and 70 nm to 120, 350, and 7,202 nm, respectively. This suggested the good responsiveness of the micelles to ROS. The potential mechanism is that the cleavability of TK by ROS leads to the hydrophobic drug removal from the micelle core, resulting in the hydrophobic core of micelles being transformed to hydrophilic, and then induces the disassembly of micelles.

Subsequently, the ROS-triggered drug release behavior of PCM2 was further tested in the present study. It was found that the TK linkers between drugs and DEX maintained stability and less than 5% of the drugs was released from PCM2 even after incubation for 60 h. On the contrary, about 43 and 55% of PTX and CuB, respectively, were leaked within 60 h after incubation with 0.1 mM H_2_O_2_. While PCM2 was treated with 10 mM H_2_O_2_, the cumulative release of PTX and CuB at 60 h reached 74 and 84%, respectively. These results confirmed that the concentration of H_2_O_2_ had a positive and significant influence on the amount and the rate of drugs released, which was the basement of self-amplification drug release in the tumor intracellular conditions.

### Cellular Uptake

**FIGURE 3:**
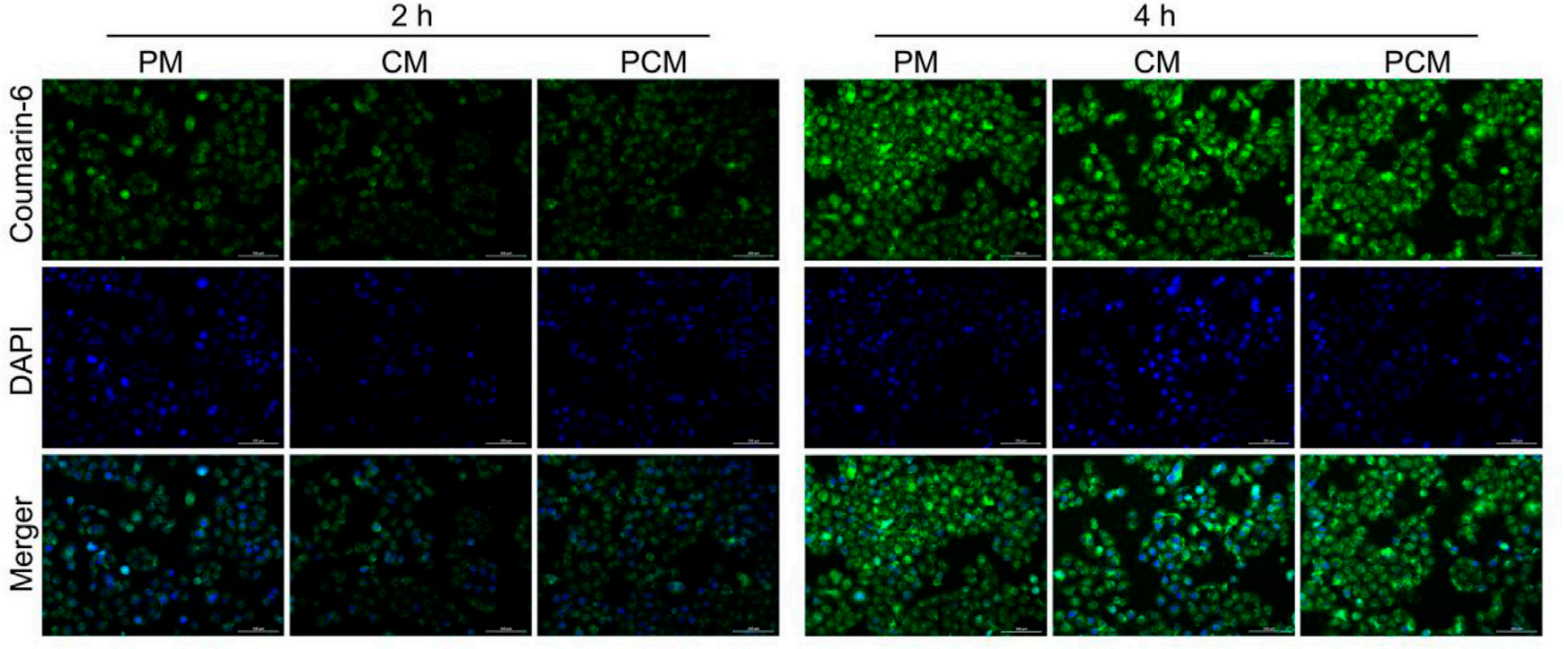
Fluorescence microscope images of BGC-823 cells after incubation with coumarin-6-loaded PM, CM, and PCM2 micelles for 2 or 4 h, respectively.

In this study the cellular internalization process of PCM2 against BGC-823 cells was monitored using a fluorescence microscope (Figure 3). After a 2 h incubation period, the weakly green fluorescence could be observed in the cytoplasm in the three micelle treatment group. This demonstrated that the micelles could effectively be internalized by BGC-823 cells. When the incubation time was increased to 4 h, the green fluorescence signal in the three micelle group was also increased, and this suggested the time-dependent cellular uptake. In addition, it was found that the fluorescence intensity had no significant difference within the same incubation period. This indicated that the three kinds of micelles could be effectively internalized by cancer cells and there was no remarkable difference in the uptake.

### Reactive Oxygen Species Generation and Reactive Oxygen Species-Triggered Drug Release in Cancer Cells

In the hypothesis of the present study, the released CuB in cancer cell could induce a large amount of ROS generation, which in turn could accelerate and amplify the release of drug. To confirm this feature, the PCM intracellular ROS production capacity of different formulations was determined using DCFA-DA as the probe. It was evident that the cell-permeable nonfluorescent DCFA-DA could be easily and quickly oxidized to dichlorofluorescein (DCF) with green fluorescence using the intracellular ROS. First, the time- and dose-dependent ROS production in human gastric cancer BGC823 cells after treatment with free CuB were quantified through flow cytometry. It was found that the mean fluorescence intensity (MFI) in CuB-treated BGC823 cells was enhanced by increasing the incubation time or treatment dose (Supplementary Figures S4A,B). This demonstrated that the CuB could effectively induce ROS generation in BGC823 cells.

**FIGURE 4:**
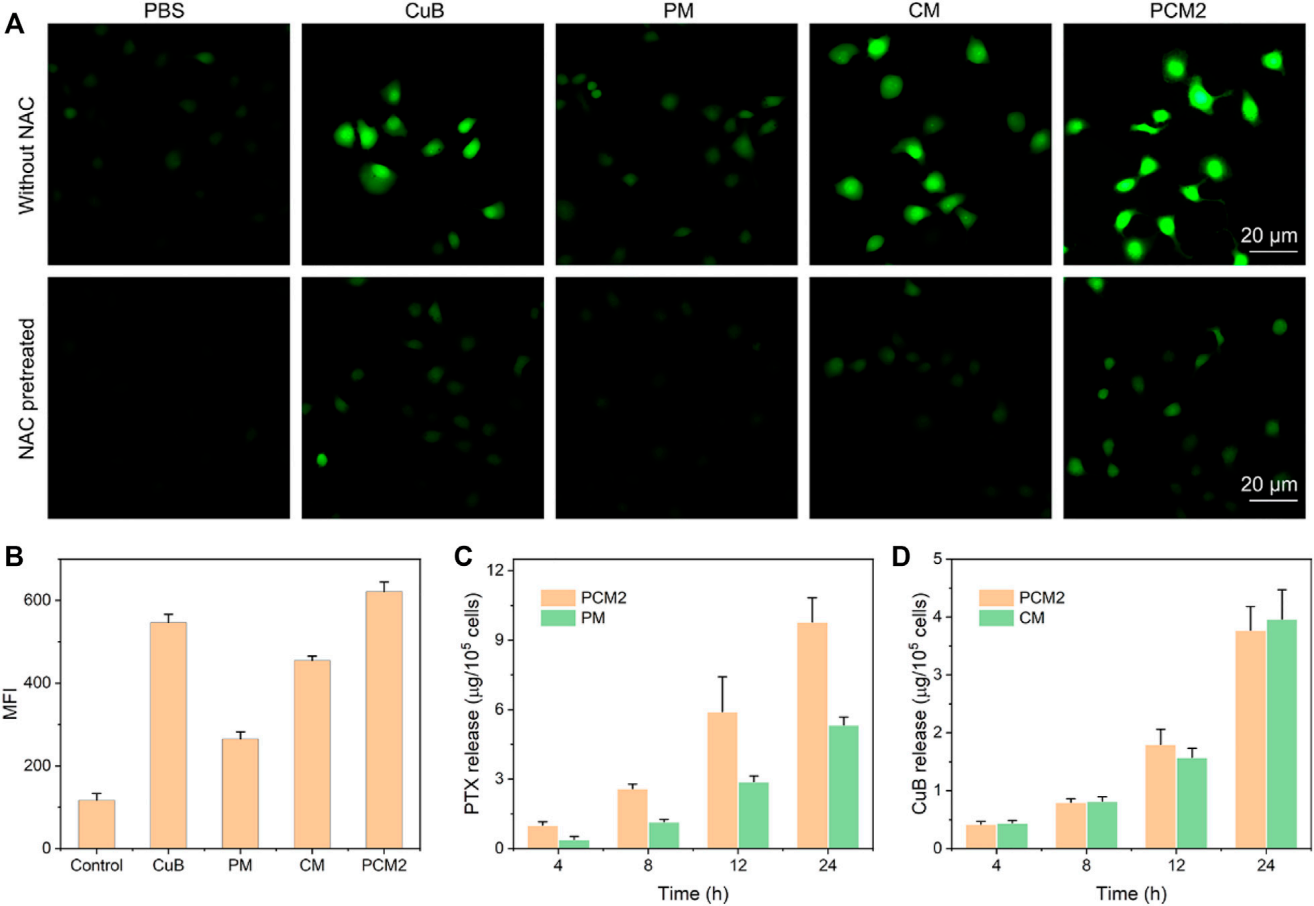
Intracellular ROS generation. **(A)** Fluorescence microscopic images of BGC823 cells after treatment with PBS, CuB, CM, PM, or PCM2 for 4 h equal to 1 μg/ml of CuB with or without NAC pretreatment. **(B)** Mean fluorescence intensity (MFI) of BGC823 cells after being treated with free CuB, CM, PM, or PCM2 (equal to 1 μg/ml of CuB) for 4 h **(C,D)** Intracellular release of PTX after incubation with PM and PM2 **(C)** and intracellular release of CuB after incubation with CM and PM2 **(D)**. Data in **(B–D)** were exhibited as the mean ± SD, *n* = 3.

Therefore, the ROS regeneration potential of CM, PM, and PCM2 against BGC823 cells was further observed by using a fluorescence microscope and quantified through flow cytometry. Furthermore, it was found that a stronger green fluorescence could be observed in CM-treated cells which demonstrated that the CM could effectively induce ROS production in cancer cells as compared with the control group (Figure 4A). Notably, it was found that the PM-treated cells showed slightly enhanced fluorescence intensity as compared with cells in the control group and this could have been induced by the released PTX. The qualitative results show that the PCM2-treated cells exhibit a stronger fluorescence intensity compared with PM and CM under the synergy of CuB and PTX. Furthermore, the results of fluorescence microscopy were well consistent with those of flow cytometry (Figure 4B). Moreover, the generated ROS in CuB, CM, and PCM2 groups can be scavenged by the ROS scavenger, NAC, further demonstrating CuB could effectively trigger ROS generation in cancer cells. Therefore, the results of this study demonstrated that the CuB-based formulations can effectively promote ROS generation in cancer cells.

To further verify whether the CuB-mediated ROS production could also promote drug release, the released PTX and CuB in CM (17 μg/ml of CuB)-, PM (50 μg/ml of PTX)-, and PCM2 (17 μg/ml of CuB and 50 μg/ml of PTX)-treated cells were quantified using HPLC. It was found that the released drugs in each formulation were increasing with the extension of incubation time (Figures 4C,D). The release of PTX in the PCM2-treated group at the incubation period of 4, 8, 12, and 24 h was 2.6-, 2.2-, 2.1-, and 1.8-fold higher than that of PM, respectively. However, the release of CuB in the CM- and PCM2-treated group at the same incubation period had no remarkable difference. These results suggested that CuB can effectively promote the drug release in cancer cells.

### *In Vitro* Cytotoxicity Assay

**FIGURE 5:**
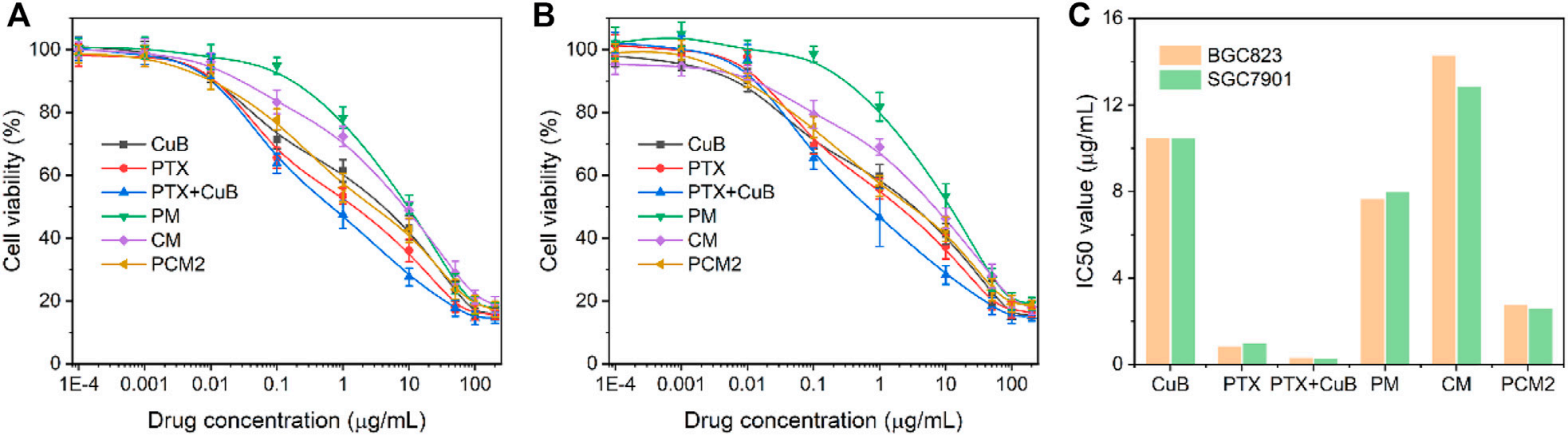
*In vitro* antitumor effect. Cell cytotoxicity **(A,B)** and IC_50_ value **(C)** of CuB, PTX, PTX+CuB, PM, CM, and PCM2 against BGC823 **(A)** and SGC7901 **(B)** cells at 48 h. Data in **(A,B)** are shown as the mean ± SD, *n* = 6.

The cytotoxicity of different drug formulations was evaluated in the current study using BGC823 cells and SGC7901 cells through the MTT method and the IC_50_ values were simultaneously calculated (Figures 5A–C). Results of this study show that both free CuB and CMs had a weak cell-killing ability with a high IC_50_ value, which was 10.52/10.49 μg/ml and 14.33/12.87 μg/ml in BGC823 cells and SGC7901 cells, respectively. Furthermore, it was found that the IC_50_ value of PM was 7.69 and 8.0 μg/ml in BGC823 cells and SGC7901 cells at 48 h, which was lower than that of PTX due to the delayed PTX release. This was because PTX’s main antitumor active group C2′-hydroxyl was blocked (Wang et al., 2020). On the contrary, it was found that PCM2 exhibited a better cytotoxicity in comparison with PTX and showed a similar cancer cell-killing capability in comparison with the combination of PTX and CuB. The results of this study show that the IC_50_ value of PCM2 was 2.78/2.63 μg/ml in comparison with 0.87/1.00 μg/ml for PTX and 0.33/0.29 μg/ml for the combination of PTX and CuB (equal to PTX) against BGC823 cells and SGC7901 cells, respectively. Subsequently, to evaluate the synergistic effect of CuB and PTX, the combination index (CI) was calculated according to the Chou-Talalay equation (Li et al., 2020): CIx = D_1_/D_x1_ + D_2_/D_x2_. In the present study, D_x1_ and D_x2_ are represented as the IC_50_ value of PTX and CuB alone, respectively. The D_1_ and D_2_ were defined as the dose of PTX and CuB in the co-treatment group at the IC_50_ value. Furthermore, the CI > 1, CI = 1, and CI < 1 were denoted as antagonism, additive effect, and synergism, respectively. The CI_50_ value of PCM2 against BGC823 cells and SGC7901 cells was 0.39 and 0.30 which demonstrated a strong synergic effect and good sequential prodrug bioactivation.

### *In Vivo* Antitumor Efficacy

**FIGURE 6:**
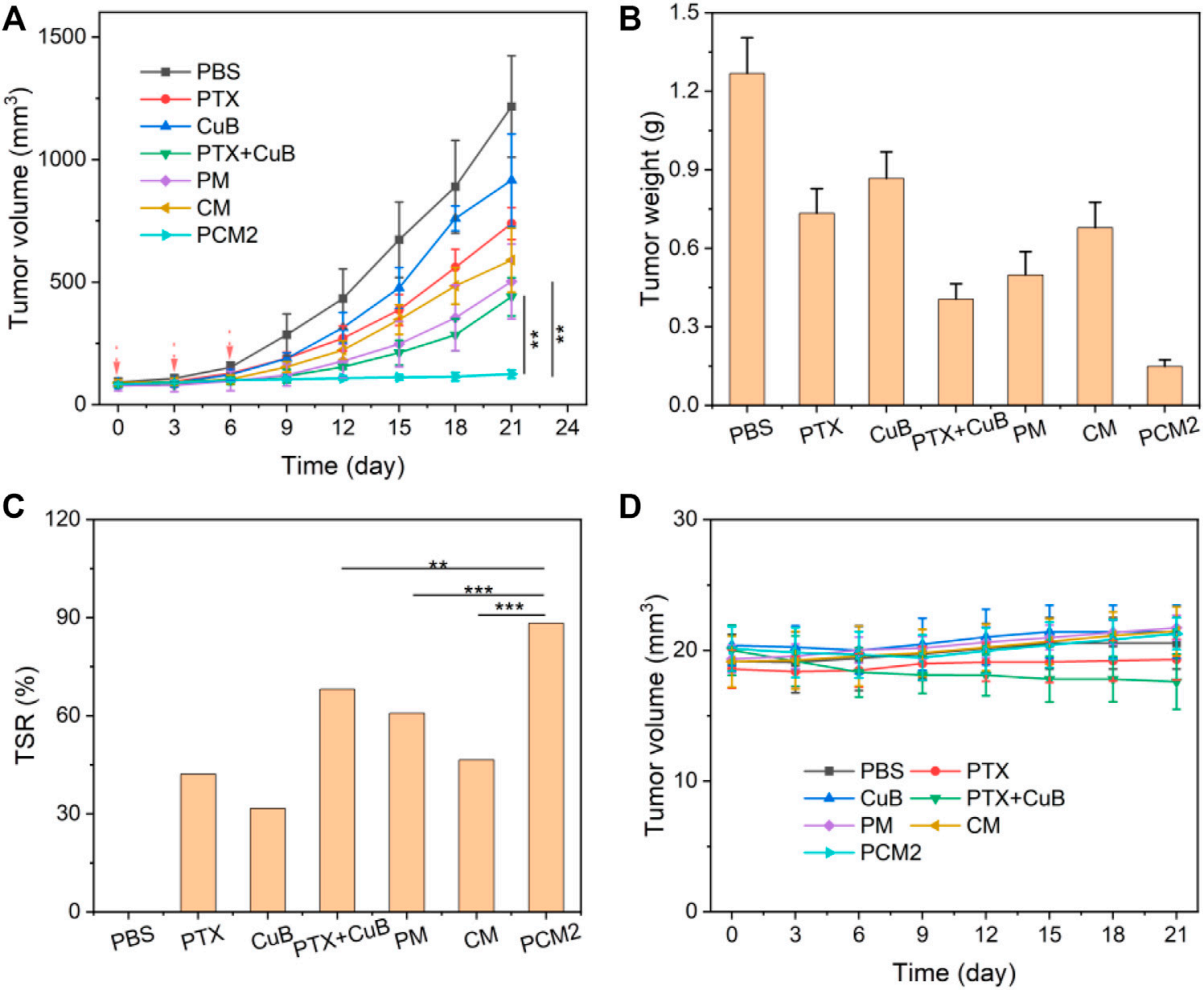
*In vivo* antitumor effects. **(A)** Changes in tumor volume during the treatment cycle. **(B)** Dissected tumor weight at day 21 in different groups. **(C)** TSR of PTX, CuB, PTX+CuB, PM, CM, and PCM2. **(D)** Changes in body weight during the treatment cycle. Data are exhibited as the mean ± SD, *n* = 5.

The *in vivo* anticancer effects of PCM2 were further investigated using BGC823 tumor-bearing mice. As shown in Figures 6A–C, the tumor volume of mice treated with saline rapidly increased from 100 mm^3^ to about 1,216 mm^3^ within 21 days. For mice treated with PTX, CuB, and CM, tumor volume increased to 739 mm^3^, 916 mm^3^, and 590 mm^3^ at the end of 21 days of treatment. The TSR of PTX, CuB, and CM was 42.1, 31.6, and 46.4%, indicating poor antitumor effect. The tumor volume of mice treated with a combination of PTX and CuB was 440 mm^3^, and the TSR was 68.0%. This may be due to poor solubility, low drug accumulation in tumors, and short duration in blood circulation (Sauraj et al., 2018). In contrast, the tumor volume of mice treated with PM was moderately reduced to about 503 mm^3^ on day 21, and its TSR was 60.7%. Notably, among the evaluated formulations, PCM2 exhibited the best antitumor effect, with a tumor size of as low as ∼125 mm^3^ for the same therapeutic period and its TSR as high as 88.3%. The high antitumor effect was ascribed to the rapid on-site prodrug bioactivation triggered by ROS generated in response to CuB. Changes in bodyweight were recorded to reflect the safety of PCM2. As displayed in Figure 6D, the bodyweight of mice administered with free PTX or free CuB was slightly higher compared to that of mice that received PTX+CuB treatment within 21 days. Contrarily, no remarkable body weigh lost was discovered in mice treated with micelle formulations, suggesting that PCM2 was safe.

## Conclusion

In summary, an ROS-responsive, self-amplification drug release nanococktail (PCM) was successfully developed in the present work by co-assembling an ROS-sensitive PTX prodrug and CuB prodrug. The nanococktail was effectively uptaken by tumor cells and induced intracellular ROS generation which further enhanced drug release. The developed PCM inhibited the growth of tumor cells *in vitro* and *in vivo* without causing significant systemic toxicity. Overall, polymeric-prodrug-based nanococktails with self-amplification drug release possess good antitumor effects and have low drug-related toxicity.

## Data Availability

The original contributions presented in the study are included in the article/Supplementary Material, further inquiries can be directed to the corresponding author.

## Glossary

CI: combination index
CM: CuB loaded micelles
CMC: critical micelle concentration
CuB: cucurbitacin B
DCF: dichlorofluorescein
DCFH-DA: 2,7-dichlorodihydrofluorescein diacetate
DEX: dextran
DEX-TK-CuB: CuB polymeric prodrug
DEX-TK-PTX: PTX polymeric prodrug
DLC: drug loading content
DLS: dynamic light scattering
DMAP: 4-dimethylaminopyridine
DMSO: dimethylsulfoxide
EDC: 1-(3-Dimethylaminopropyl)-3-ethylcarbodiimide hydrochloride
EPR effect: enhanced permeability and retention effect
FBS: fetal bovine serum
GC: gastric cancer
HPLC: high performance liquid chromatography
IC50: half maximal inhibitory concentration
MS: mass spectrometer
MW: molecular weight
MWCO: molecular weight cut off
NAC: N-acetyl cysteine
PBS: phosphate buffer
PCM: PTX and CuB co-loaded micelles
PM: PTX loaded micelles
PTX: paclitaxel
ROS: reactive oxygen species
TEM: transmission electron microscopy
TK: thioketal linker
TK-CuB: the conjugation of TK and CuB
TK-PTX: the conjugation of TK and PTX
TSR: tumor suppression ration
