# Supplementary material for: Reactive Oxygen Species-Responsive Nanococktail With Self-Amplificated Drug Release for Efficient Co-Delivery of Paclitaxel/Cucurbitacin B and Synergistic Treatment of Gastric Cancer
Source: Front Chem. 2022 Mar 4;10:844426. doi: 10.3389/fchem.2022.844426 (PMC8931329; doi:10.3389/fchem.2022.844426)
Supplement: Supplementary file 1 [file DataSheet1.docx]

**ROS-responsive nanococktail with self-amplificated drug release for efficient co-delivery of paclitaxel/cucurbitacin B and synergistic treatment of gastric cancer**

Lijun Pang^1^, Lei Zhang^2^, Hong Zhou^1^, Ling Cao^1^, Yueqin Shao^1^, Tengyun Li^2^

1. Department of Oncology, Jiangsu Shengze Hospital Affiliated with Nanjing Medical University, Suzhou 215228, China

2. Department of Pharmacy, Jiangsu Shengze Hospital Affiliated with Nanjing Medical University, Suzhou 215228, China

**Corresponding author:**

Tengyun Li,

Address: Department of Pharmacy, Jiangsu Shengze Hospital Affiliated with Nanjing Medical University, Suzhou 215228, China

Email: 123150774@qq.com

**Supporting experiments**

**1. Materials**

Dextran (Dex, *Mn* = 70 kDa) used in the present study was acquired from Sigma-Aldrich (St. Louis, MI, USA). The paclitaxel (PTX), cucurbitacin B (CuB), 1-(3-Dimethylaminopropyl)-3-ethylcarbodiimide hydrochloride (EDC), CH_2_Cl_2_, dimethyl sulfoxide (DMSO), and 4-dimethylaminopyridine (DMAP) were purchased from Aladdin Reagents (Shanghai, China). The 3-(4,5-Dimethythiazol-2-yl)-2,5-diphenyltetrazolium bromide (MTT), 2,7-dichlorodihydrofluorescein diacetate (DCFH-DA), and Hoechst 33342 were purchased from Beyotime Biotech Co., Ltd (Shanghai, China).

**2. Characterizations**

The surface charge and size as well as the size distribution (polydispersity index, PDI) of micelles were determined using the dynamic light scattering (DLS), recorded on a Nano-Zetasizer (Malvern, UK). Hitachi HT7800 transmission electron microscopy (TEM, Hitachi, Japan) was used to image the morphology of micelles

High performance liquid chromatography (HPLC) analysis was carried out using a Shimadzu HPLC system (Shimadzu, Kyoto, Japan) which was equipped with a reversed-phase column (Hypersil BDS C18 5 μm, 4.6 mm × 250 mm, Dalian Elite Analytical Instruments CO., Ltd., China), a LC-20A pump, and SPD-20A UV detector. To detect the PTX, the mobile phase was prepared with a mixture of acetonitrile and water (60: 40, v/v), the flow rate was 1.0 mL/min, and the detector wavelength was set at 227 nm. The HPLC was calibrated using standard solutions of 0.01-50 μg/mL of PTX dissolved in methanol (correlation coefficient of R^2^ = 0.9981).

To detect the CuB, the detector wavelength was set at 293 nm whereas the mobile phase was prepared using a mixture of methanol/water (70/30, v/v) and the flow rate was 1 mL/min. The HPLC was calibrated with standard solutions of 0.01-50 μg/mL of CuB dissolved in methanol for CuB detection (correlation coefficient of R^2^ = 0.9972).

**3. Cell culture and animals**

Human gastric cancer BGC-823 cells were obtained from the National Collection of Authenticated Cell Cultures (Shanghai, China) and were cultured in DMEM supplemented with 10% FBS under an atmosphere of 5% CO_2_.

Male BALB/c-nude mice (5-6 weeks, 18 ± 2 g) were obtained from the Experimental Animal Center of Nanjing Medical University. The experimentation protocols for all animal studies were approved by the Animal Care and Use Committee of the Nanjing Medical University.

**4. Critical micelle concentration (CMC) detection**

A stock solution of prodrugs (2 mg/mL) was prepared in PBS with sonication. A known amount of Nile Red in CH_2_Cl_2_ was added to a series of vials and was then evaporated. Subsequently, a measured amount of stock solution was added into each vial, followed by PBS to increase the concentration from 5 × 10^-4^ to 2 mg/mL. The final concentration of Nile Red was fixed at 1 ×10^-6^ M. The vials were stirred overnight at room temperature to equilibrate the Nile Red with the micelles and their fluorescence intensity was then recorded. Using a graph of fluorescence intensity vs. log (prodrug concentration), the CMC was determined as the intersection of the tangents to the two linear portions of the graph.

**5. *In vitro* stability**

The stability of micelles was determined by monitoring they size changes. Briefly, fresh prepared micelles dispersed by PBS with or without 10% FBS to obtain 2 mg/mL micelles solution. The micelles solution was cultured at 37 °C with slight stirring. The size of micelles was detected by DLS at the pre-set time points.

**6. Intracellular ROS-triggered drug release**

The BGC823 cells were seeded 60 mm dish with 2 × 10^5^ cells per dish and cultured overnight. Then, cells were treated with CM, PM, and PCM (equal to 12 μg/mL of PTX and 4 μg/mL of CuB) for 8, 12, or 24 h, respectively, at 37 °C. The cells were then washed with cold PBS for three times and transferred to a 10 mL centrifuge tube. The tube was placed in thermostatic ice bath at 0 °C and then sonicated the cell suspension with alternative cycles of 5 s pulses after every 10 s intervals for 5 mins using ultrasonicator probe (VCX 2500). The released PTX or CuB was then extracted using methanol/chloroform (30:70, v/v). The lysates were collected and centrifuged at 8,000 g for 10 min and at 4 °C to obtain the supernatant. The concentration of PTX and CuB in the supernatant was measured using the HPLC.

**Supporting figures**





**Scheme S1.** The synthesis routes or TK anhydride (A), TK-PTX (B), TK-CuB (C), and DEX-TK-CuB and DEX-TK-PTX.


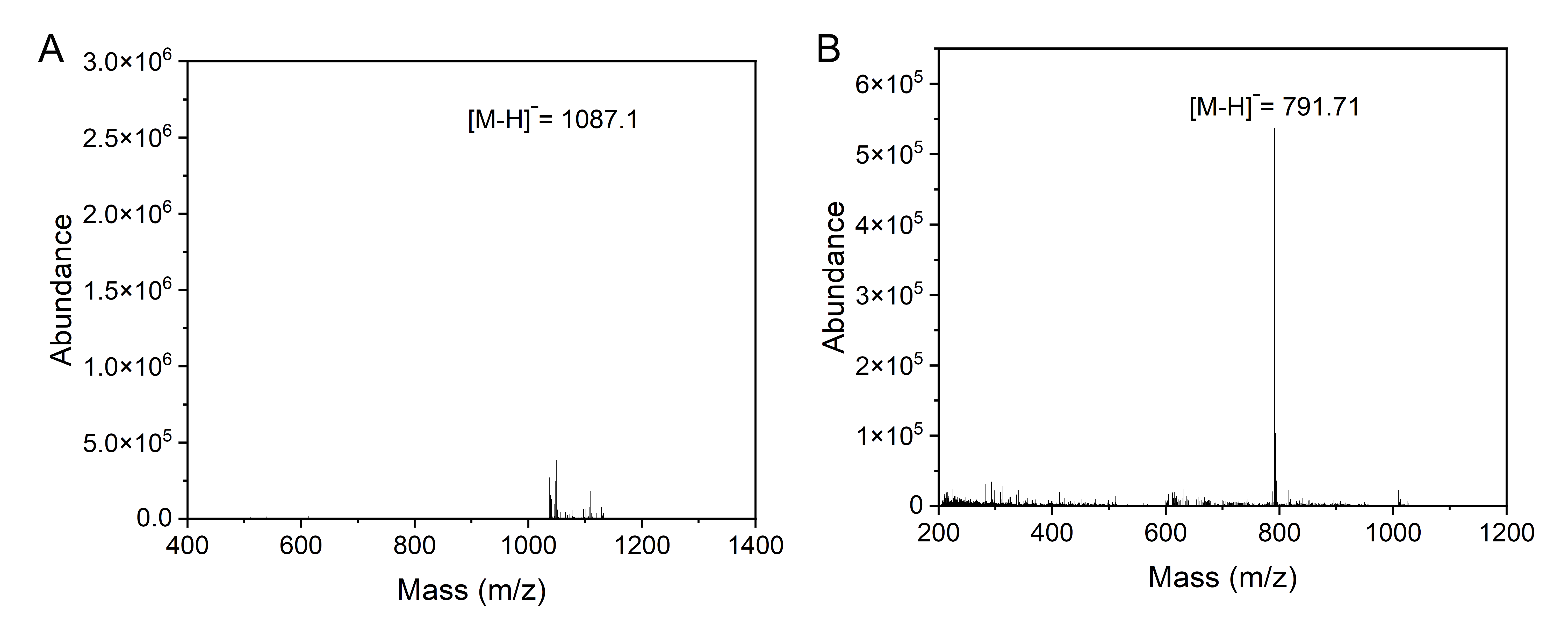


**Fig. S1** Mass spectrums of TK-PTX (A) and TK-CuB (B).


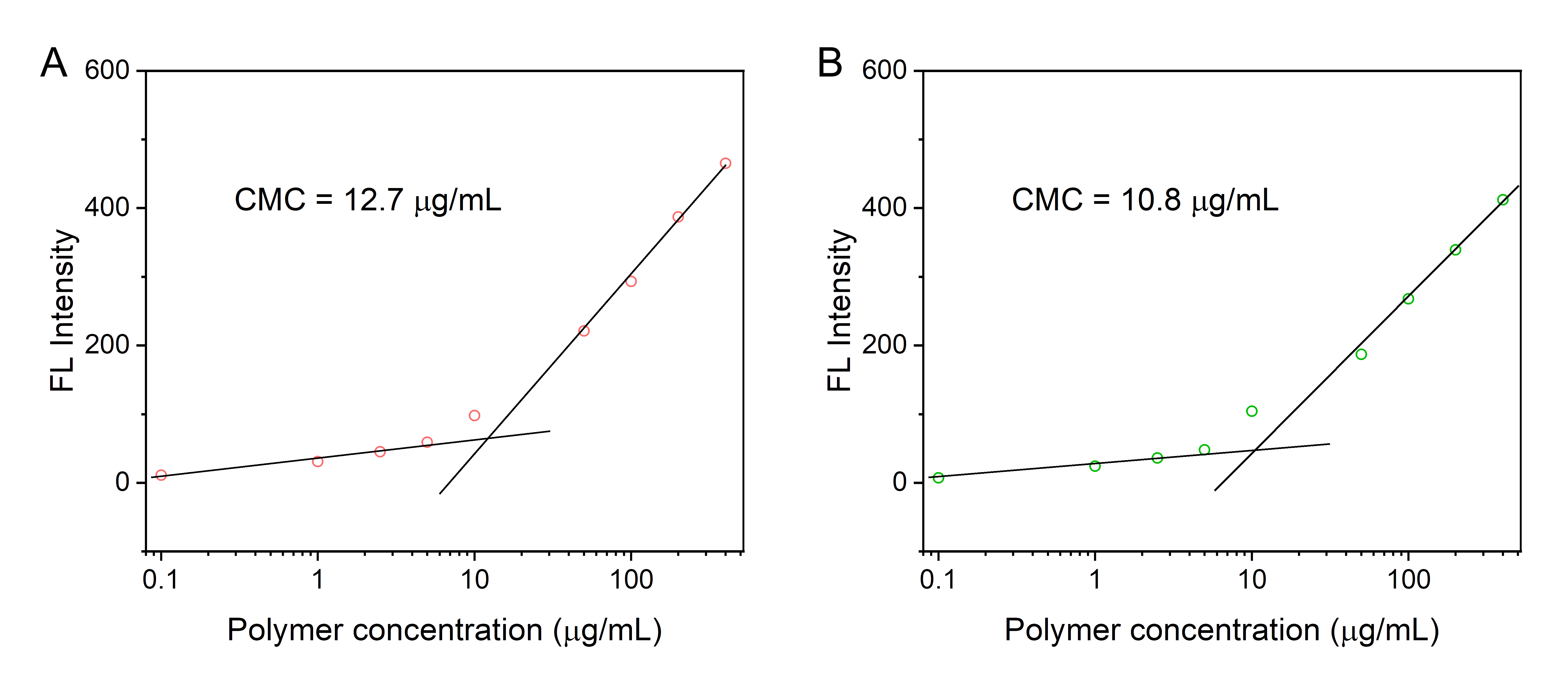


**Fig. S2** CMC of DEX-TK-PTX (A) and DEX-TK-CuB (B).


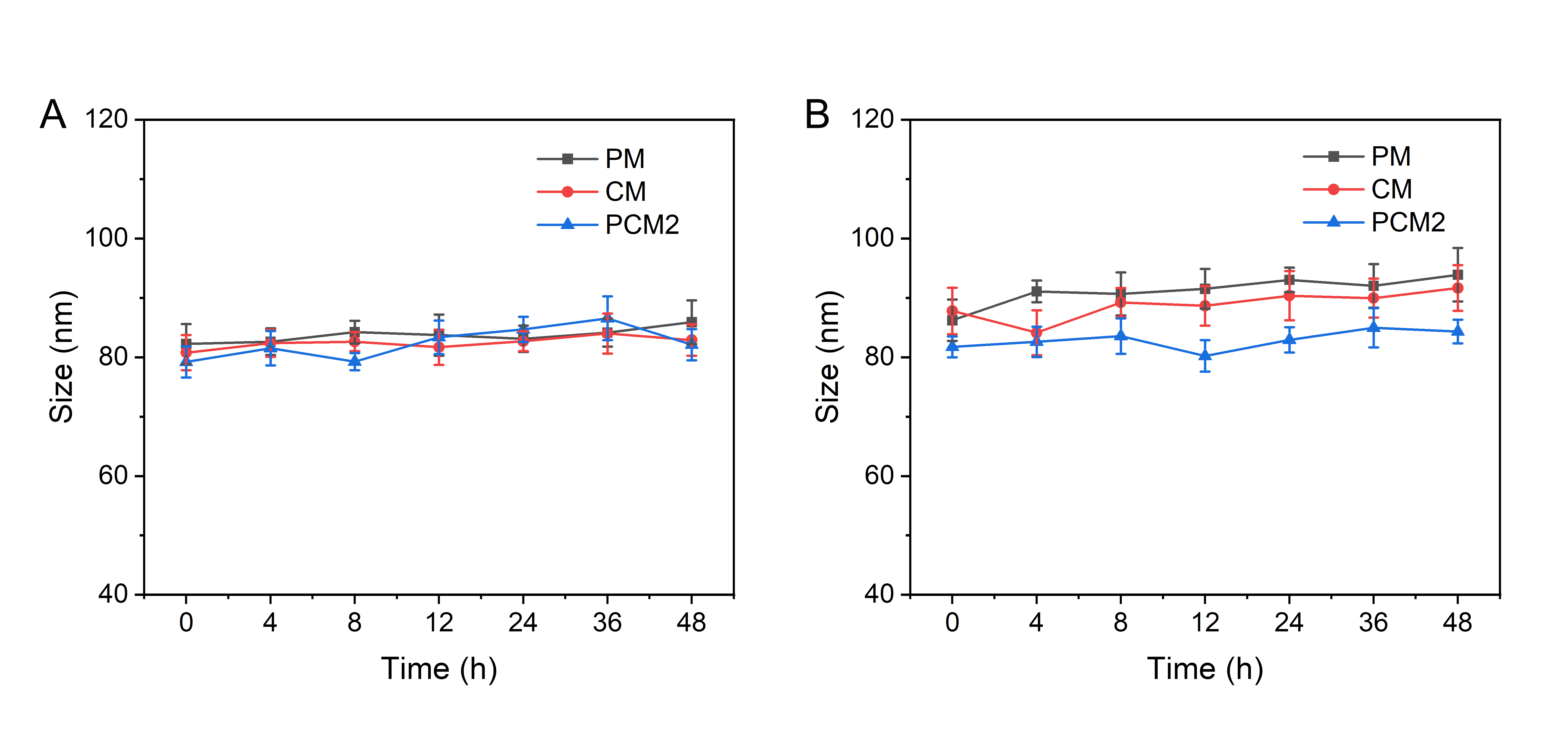


**Fig. S3** Stability assay. Size changes of PM, CM, and PCM2 after incubated in PBS (pH 7.4) (A) or PBS (pH 7.4) containing 10% FBS (B).


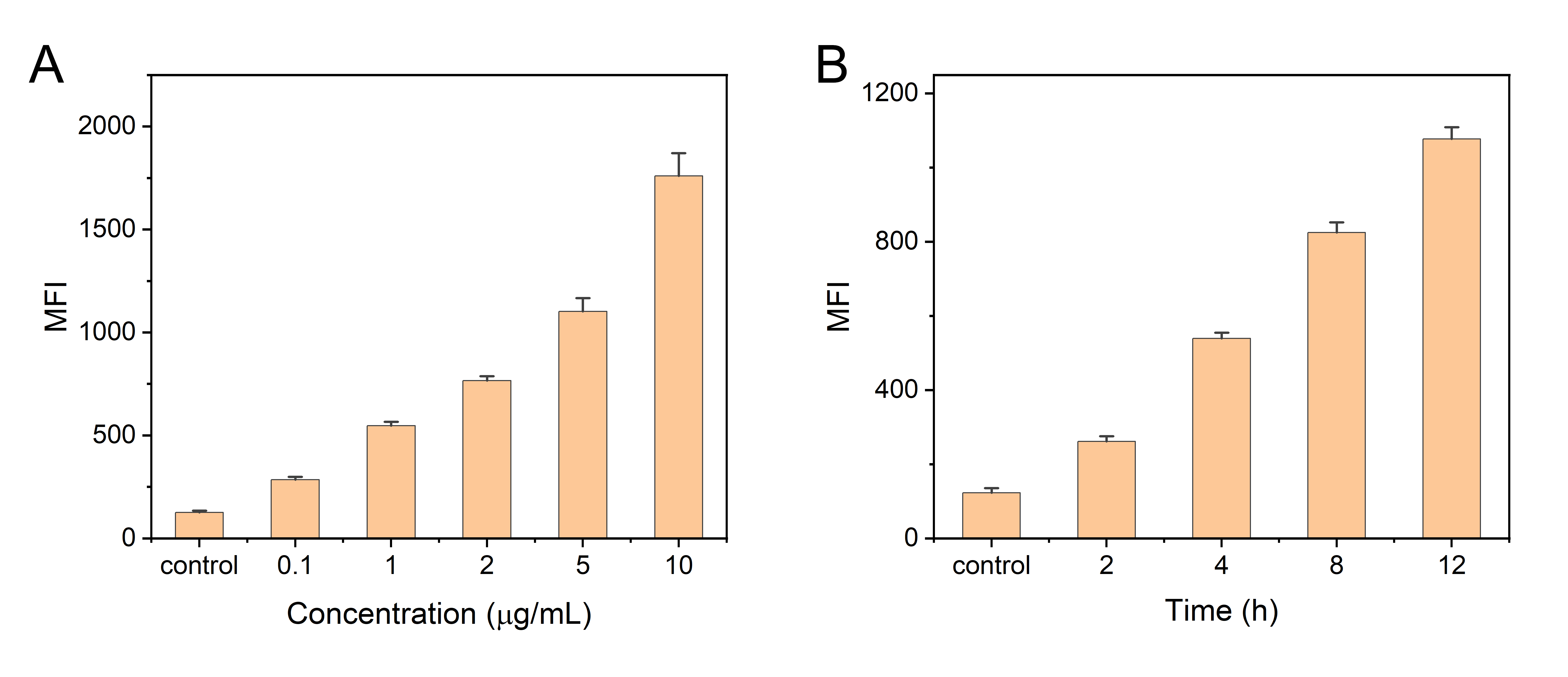


**Fig. S4** CuB mediate ROS generation in BGC823 cells. MFI of DCFH in cells after treated with CuB at various concentration (A) or incubated with 1 µg for different times (B). Data shown as mean ± SD, *n* = 3.

Supporting tables

| **Table S1.** Characterizations of different PCM micelles. | | | | | |
| --- | --- | --- | --- | --- | --- |
| Micelles | PTX/CuB (mass ratio) | Size (nm) | PDI | Zeta potential (mV) | IC_50_ (µg/mL) |
| PM | 1/- | 82.2 ± 3.4 | 0.275 ± 0.043 | -17.2 ± 1.0 | 7.67 |
| CM | -/1 | 80.8 ± 3.0 | 0.245 ± 0.044 | -18.5 ± 1.3 | 14.33 |
| PCM1 | 5/1 | 82.3 ± 3.6 | 0.268 ± 0.050 | -17.6 ± 1.8 | 7.15 |
| PCM2 | 3/1 | 78.7 ± 2.5 | 0.253 ± 0.026 | -17.4 ± 1.8 | 2.78 |
| PCM3 | 1/1 | 79.0 ± 2.6 | 0.291 ± 0.040 | -17.0 ± 1.5 | 4.57 |
| PCM4 | 1/3 | 80.0 ± 3.6 | 0.267 ± 0.016 | -16.7 ± 1.4 | 4.24 |
| PCM5 | 1/5 | 96.7 ± 3.2 | 0.308 ± 0.029 | -17.6 ± 1.3 | 3.16 |
